# A multi-epitope pan-betacoronavirus vaccine construct predicted to induce broad-spectrum and durable immune responses: an immunoinformatics approach

**DOI:** 10.3389/fbinf.2026.1784011

**Published:** 2026-03-18

**Authors:** Anabella Margareth Arapa, Trina Ekawati Tallei, Rinaldi Idroes, Fatimawali, Elly Juliana Suoth, Maghfirah Savitri, Ahmad Akroman Adam, Beivy Jonathan Kolondam, Chika Yamada, Rosy Iara Maciel de Azambuja Ribeiro, Amama Rani, Moon Nyeo Park, Youdiil Ophinni, Bonglee Kim

**Affiliations:** 1 Pharmacy Study Program, Faculty of Mathematics and Natural Sciences, Sam Ratulangi University, Manado, North Sulawesi, Indonesia; 2 Department of Biology, Faculty of Mathematics and Natural Sciences, Sam Ratulangi University, Manado, North Sulawesi, Indonesia; 3 Department of Biology, Faculty of Medicine, Sam Ratulangi University, Manado, North Sulawesi, Indonesia; 4 Department of Pathology, College of Korean Medicine, Kyung Hee University, Seoul, Republic of Korea; 5 Department of Pharmacy, Faculty of Mathematics and Natural Sciences, Universitas Syiah Kuala, Banda Aceh, Indonesia; 6 Robert Wolter Monginsidi Army Hospital, Manado, North Sulawesi, Indonesia; 7 Cosmo Dental Care, Manado, North Sulawesi, Indonesia; 8 Center for Southeast Asian Studies (CSEAS), Kyoto University, Kyoto, Japan; 9 Experimental Pathology Laboratory, Midwest Campus, Federal University of São João del-Rei, Divinópolis, Brazil; 10 The Hakubi Center for Advanced Research, Kyoto University, Kyoto, Japan; 11 Division of Clinical Virology, Center for Infectious Diseases, Graduate School of Medicine, Kobe University, Kobe, Japan; 12 Department of Host Defense, Immunology Frontier Research Center (IFReC), Osaka University, Osaka, Japan

**Keywords:** conserved epitopes, immune simulation, immunoinformatics, multi-epitope peptide vaccine, pan-betacoronavirus, receptor-binding domain (RBD), reverse vaccinology, β-defensin adjuvant

## Abstract

**Introduction:**

Recurrent zoonotic spillovers and continuous antigenic evolution among betacoronaviruses, including SARS-CoV, MERS-CoV, and SARS-CoV-2, highlight the urgent need for a broad-spectrum vaccine capable of eliciting cross-protective immunity. Conventional vaccines, although effective against specific strains, may be limited by antigenic mismatch and waning immunity. This study aimed to design a multi-epitope pan-betacoronavirus vaccine targeting conserved regions within the receptor-binding domain (RBD) using an integrated immunoinformatics and reverse vaccinology framework.

**Methods:**

Cytotoxic T-lymphocyte (CTL), helper T-lymphocyte (HTL), and linear B-cell epitopes were predicted and screened for antigenicity, allergenicity, toxicity, and non-homology to host proteins. Selected epitopes were assembled into a 285–amino acid multi-epitope construct using optimized linkers (AAY, GPGPG, EAAAK, and GGGGS) and human β-defensin 3 as an adjuvant. Structural modeling and refinement were performed to generate a three-dimensional vaccine model, followed by molecular docking with a B-cell receptor (BCR) Fab model using ClusPro. Molecular dynamics simulations were conducted to evaluate structural stability, and immune responses were assessed through computational immune simulation.

**Results:**

The refined vaccine construct produced a stable structural model with a C-score of −3.60. Molecular docking identified a highly ranked complex from a well-populated cluster (Cluster 1; 49 members) with a Lowest Energy score of −865.9, indicating favorable interface complementarity under the docking scoring function. Molecular dynamics simulation over 100 ns supported the structural integrity and dynamic stability of the complex, with minimal backbone deviation and sustained intermolecular interactions. Immune simulations predicted coordinated humoral and cellular responses following a simulated prime–boost regimen, including increased antibody titers, elevated IL-2 and IFN-γ levels, and sustained memory B- and T-cell populations. The selected epitope set showed an estimated global HLA population coverage of 93.28%.

**Conclusion:**

This study identifies a promising *in silico* multi-epitope RBD-based pan-betacoronavirus vaccine candidate with predicted broad HLA population coverage and favorable structural stability. These findings provide a computational basis for subsequent experimental validation of the construct’s immunogenicity, safety, and potential cross-protective capacity in relevant *in vitro* and *in vivo* models.

## 1 Introduction

Since 2002, at least three highly pathogenic betacoronaviruses (SARS-CoV, MERS-CoV, and SARS-CoV-2) have spilled over into humans (Al-Salihi and Khalaf, 2021), while four endemic “common cold” human coronaviruses (HCoVs), HCoV-229E and HCoV-NL63 (Alphacoronavirus) and HCoV-OC43 and HCoV-HKU1 (Betacoronavirus), continue to circulate globally and cause recurrent respiratory infections. The ongoing evolution of SARS-CoV-2, particularly the emergence of Omicron-lineage subvariants driven by antigenic drift and recombination, has necessitated multiple vaccine updates. Omicron-era SARS-CoV-2 lineages contain numerous RBD substitutions associated with immune escape and reduced neutralization (Abbad et al., 2025), Together with the persistent circulation of HCoVs, underscores the need for vaccines that can provide broad, cross-species and cross-variant protection rather than narrow strain-specific immunity. Recent reviews have emphasized both the feasibility and urgency of developing pan-betacoronavirus vaccines to mitigate the risk of future zoonotic spillovers and variant-driven outbreaks (Tang et al., 2022; Liu et al., 2021; Jo et al., 2022).

Despite a reduction in global testing and surveillance compared to earlier pandemic phases, SARS-CoV-2 remains a persistent global threat associated with significant ongoing mortality. World Health Organization (WHO) data indicate that deaths were still being recorded across multiple geographic regions as recently as late 2025 and into early 2026 (WHO, 2026). Furthermore, sporadic zoonotic MERS-CoV infections continue to emerge, highlighting the persistent risk of coronavirus spillover from animal reservoirs (WHO, 2025). In parallel, the endemic seasonal HCoVs (229E, NL63, OC43, HKU1) maintain a consistent background of transmission, contributing substantially to the global burden of respiratory infections (Siragam et al., 2024).

The trajectory of COVID-19 vaccination, which progressively expanded to encompass all age and risk groups (Hong et al., 2024), demonstrated the high initial efficacy of first-generation strain-specific formulations, including mRNA, viral vector, inactivated, and subunit platforms. However, the neutralizing breadth of these vaccines against emerging variants has significantly eroded, largely due to antigenic mismatch and waning immunity. Even updated boosters, which target a limited number of variants, often lag behind the rapid pace of viral evolution. These limitations have prompted the development of variant-agnostic and cross-lineage vaccine strategies, alongside interest in bioactive natural compounds with antiviral or immunomodulatory properties. Phytochemicals such as flavonoids have been shown to inhibit HIF-1α–mediated inflammation (Han et al., 2024; Vegad et al., 2024) and interfere with ACE2–spike interactions (Lee et al., 2025), suggesting their potential as adjunctive interventions in coronavirus control.

Although no true pan-coronavirus vaccine has yet reached licensure, several broad-spectrum or pan-sarbecovirus candidates are in development, spanning computational design and preclinical evaluation. For example, the CD40.CoV2 vaccine, a pan-sarbecovirus construct, showed immunogenicity and antiviral efficacy in animal models (Coléon et al., 2021). Multiantigen DNA vaccines targeting conserved structural and nonstructural regions across sarbecoviruses (CoVAX_MNS and CoVAX_ORF1ab) elicited robust T-cell responses and partial protection in hACE2-transgenic mice (van Bergen et al., 2023). Furthermore, computational antigen design has advanced: novel in silico–engineered Spike variants eliciting broad neutralization across variants of concern (VOCs) were demonstrated in animal immunization platforms (Vishwanath et al., 2024). Even at the receptor-binding domain (RBD) or spike domain level, rational design strategies now strive for breadth beyond individual lineages (Vishwanath et al., 2023). The feasibility, challenges, and strategies for pan-coronavirus vaccines have been extensively discussed in a separate review (Cankat et al., 2023). Yet, many of these candidates remain at early developmental stages, lacking full epitope-level optimization, broad population coverage assessment, or immune simulation pipelines.

Reverse vaccinology (RV) inverts the classical antigen-first paradigm by mining pathogen genomes or proteomes to prioritize vaccine candidates *in silico* before experimental validation. By integrating sequence conservation, antigenicity scoring, human leukocyte antigen (HLA) binding prediction, structural modeling, docking, and immune simulations, RV and immunoinformatics accelerate candidate triage and reduce wet-lab burden. This cost- and time-efficient pipeline is especially apt for pandemic-preparedness applications and has been applied to viruses, bacteria, and parasites (Ong et al., 2020; Windah et al., 2023). Compared with conventional antigen-first vaccine discovery, immunoinformatics enables rapid, systematic early-stage prioritization of conserved targets and epitope combinations while concurrently applying safety filters and estimating HLA population coverage, capabilities that are particularly valuable for breadth-oriented vaccine design and pandemic-preparedness (Basmenj et al., 2025; Rawat et al., 2023).

Multi-epitope peptide vaccine constructs leverage synergies of T- and B-cell epitopes, linked via optimal spacers and coupled to adjuvants or immunostimulatory modules (Tran et al., 2022). Such constructs aim to induce broad, multi-layered immune responses while minimizing immunodominance and immune escape (Barazani et al., 2025). While the S2 subunit of the Spike protein is more conserved and capable of inducing cross-reactive responses (Dacon et al., 2022), the RBD within the S1 subunit remains the primary neutralizing target and a focal point for host receptor interaction (Celik et al., 2022).

In this study, we focused exclusively on the RBD of the S1 subunit from SARS-CoV, MERS-CoV, and SARS-CoV-2, given its central role in viral attachment and entry. Despite its variability, conserved residues within the RBD across these pathogenic betacoronaviruses have been shown to elicit cross-reactive antibody and T-cell responses. Therefore, this study employed an immunoinformatics-guided reverse vaccinology approach to identify and integrate conserved B-cell, CTL, and HTL epitopes within the RBD sequences, constructing a multi-epitope peptide vaccine candidate with predicted cross-reactivity and broad immune coverage.

## 2 Materials and methods

### 2.1 Protein sequence collection

Protein sequences corresponding to the receptor-binding domain (RBD) of the S1 subunit from SARS-CoV, MERS-CoV, and SARS-CoV-2 were retrieved from the National Center for Biotechnology Information (NCBI) database and downloaded in FASTA format. To capture geographic and host-associated diversity, a total of 22 RBD sequences were compiled, comprising 7 SARS-CoV, 7 MERS-CoV, and 8 SARS-CoV-2 sequences, and representing multiple locations and hosts. The MERS-CoV dataset included both human and non-human isolates, such as camels and goats. All retrieved sequences were curated to remove duplicates and to ensure appropriate annotation and completeness prior to downstream analyses.

### 2.2 Multiple sequence alignment

Multiple sequence alignment (MSA) was performed on the complete dataset of 22 retrieved RBD sequences (7 SARS-CoV, 7 MERS-CoV, and 8 SARS-CoV-2) using MAFFT v7.790 implemented in Geneious Prime v2025.2.2 (Biomatters Ltd., Auckland, New Zealand). The full sequence set, including non-human MERS-CoV isolates, was explicitly retained in the alignment to capture cross-host variability and to support robust identification of conserved positions and segments. Conserved regions were defined directly from the full MSA and were subsequently used for downstream feature extraction and epitope prediction.

### 2.3 B-Cell epitope prediction

Prediction of linear B-cell epitopes was carried out using the IEDB web server (http://tools.iedb.org/bcell/) with the Bepipred Linear Epitope Prediction 2.0 algorithm (Jespersen et al., 2017; Vita et al., 2018). Epitopes within the range of 5–30 amino acids and located within conserved regions of the aligned sequences were selected for further analysis (Jespersen et al., 2017).

### 2.4 T-Cell epitope identification and immunological characterization

Cytotoxic T-lymphocyte (CTL) and helper T-lymphocyte (HTL) epitopes were identified using the Immune Epitope Database (IEDB) prediction tools. CTL epitopes were predicted through the major histocompatibility complex (MHC) class I binding prediction module (http://tools.iedb.org/mhci/) employing the NetMHCpan 4.1 EL algorithm (Andreatta and Nielsen, 2016; Reynisson et al., 2020). Peptides with a percentile rank ≤0.5% were classified as strong binders and those ≤2% as weak binders, following established conventions in epitope prediction studies (Naz et al., 2020; Pandey et al., 2018; Dhanda et al., 2013). Similarly, HTL epitopes were predicted using the MHC class II binding prediction tool (http://tools.iedb.org/mhcii/) based on the NetMHCIIpan 4.1 EL method (Reynisson et al., 2020), with peptides exhibiting percentile ranks ≤2% and ≤10% considered strong and weak binders, respectively (Zhou et al., 2025).

The immunological properties of the shortlisted T-cell epitopes were subsequently evaluated. Antigenicity was assessed using VaxiJen v2.0 (virus model) (https://www.ddg-pharmfac.net/vaxijen/VaxiJen/VaxiJen.html) at the default threshold of 0.4. Epitopes scoring ≥0.4 were considered probable antigens and prioritized. This cutoff is widely used for viral antigen prediction and corresponds to an operating point at which the VaxiJen viral model achieved ∼70% external validation accuracy (Doytchinova and Flower, 2007; Almofti et al., 2025; Kaur et al., 2024). Allergenicity was predicted using AllerTOP v2.1 (https://www.ddg-pharmfac.net/allertop_test/), which employs auto–cross-covariance transformation of amino acid E-descriptors to distinguish allergenic from non-allergenic peptides (Dimitrov et al., 2013).

Toxicity assessment was performed using ToxinPred (https://webs.iiitd.edu.in/raghava/toxinpred3/), which applies a support vector machine (SVM) classifier to differentiate toxic from non-toxic peptides (Rathore et al., 2023). Homology analysis was conducted using BLASTp (https://blast.ncbi.nlm.nih.gov/Blast.cgi) against the human proteome to exclude epitopes with significant sequence similarity to human proteins. Only non-homologous epitopes were retained for downstream analyses to minimize the risk of autoimmune cross-reactivity.

### 2.5 Population coverage analysis

Global population coverage of the selected epitopes was determined using the IEDB Population Coverage Tool (http://tools.iedb.org/population/), which estimates the theoretical coverage of HLA alleles across different ethnicities and geographic regions (Bui et al., 2006).

### 2.6 Vaccine construction

A multi-epitope vaccine construct was assembled by concatenating shortlisted CTL, HTL, and B-cell epitopes using linker sequences selected to preserve epitope independence, improve processing, and reduce junctional effects. AAY linkers were placed between CTL epitopes to promote proteasomal processing and facilitate downstream presentation of class I–restricted peptides. GPGPG linkers were used between HTL epitopes as flexible spacers that support antigen processing and MHC-II presentation, while also helping to reduce junctional immunogenicity. KK linkers were introduced between linear B-cell epitopes to aid epitope release during proteolytic processing (e.g., via reported cathepsin-associated cleavage), thereby supporting efficient epitope presentation and accessibility (Yang et al., 2025; Nonog et al., 2025; Rahman et al., 2025). To enhance immunogenicity, an N-terminal adjuvant module (β-defensin) was incorporated and separated from the epitope cassette using the rigid EAAAK linker to maintain structural segregation and minimize steric interference between functional domains (Agarwal et al., 2025). In similar immunoinformatics vaccine designs, β-defensin has been used as an immunostimulatory adjuvant to strengthen innate activation and antigen presentation, supporting more robust downstream adaptive responses (Heidarinia et al., 2025).

### 2.7 Three-dimensional vaccine model design and refinement

The tertiary structure of the vaccine construct was modeled using the I-TASSER server, and only models with a C-score between −5 and 2 were considered reliable (Lagares et al., 2020). The top-ranked model was further refined using GalaxyRefine2 (Lee et al., 2019) and ModRefiner (Xu and Zhang, 2011) to rebuild side chains, optimize hydrogen bonding, and improve overall stereochemical quality. Molecular dynamics–based refinement was applied to improve structural stability and energy minimization.

### 2.8 Molecular docking and interaction visualization

Molecular docking between the vaccine construct and an immune receptor (crystal structure of a B-cell receptor Fab fragment) was performed using the ClusPro 2.0 server. The receptor structure was retrieved from the RCSB Protein Data Bank (PDB ID: 5IFH; resolution 2.29 Å) (https://www.rcsb.org/structure/5IFH). Docking was used to assess the structural feasibility and interface complementarity of an antibody-like interaction rather than to infer antigen specificity or absolute binding affinity. Docking poses were visualized in two dimensions using PDBsum to support detailed interaction mapping. The predicted docked complexes were evaluated based on ClusPro energy-based ranking, hydrogen-bonding patterns, and key interface residues (Hessel et al., 2023).

### 2.9 Molecular dynamics simulations

The dynamic stability of the protein and protein–ligand complexes was evaluated through molecular dynamics (MD) simulations using GROMACS version 2023.1 (Abraham et al., 2015). The CHARMM General Force Field (CGenFF) was employed to parameterize the protein, while ligand topology and parameter files were generated via the SwissParam server (Zoete et al., 2011). Each system was first subjected to energy minimization *in vacuo* using the steepest descent algorithm for 2,500 steps to eliminate steric clashes and unfavorable contacts. Subsequently, the minimized structure was solvated in a cubic box filled with SPC water molecules. To neutralize the system, Na^+^ and Cl^−^ ions were added using the *gmx genion* utility until electrostatic neutrality was achieved.

Following solvation and neutralization, the systems underwent sequential equilibration under NVT and NPT ensembles. The NVT equilibration (100 ps) maintained constant particle number, volume, and temperature, gradually heating the system to 310 K. The subsequent NPT equilibration (100 ps) ensured stabilization of pressure, density, and temperature under isotropic conditions. Throughout equilibration, position restraints were applied to the protein heavy atoms to prevent large structural deviations while allowing solvent relaxation. Temperature and pressure were controlled using the v-rescale thermostat and Parrinello–Rahman barostat, respectively (Ke et al., 2022).

All covalent bonds involving hydrogen atoms were constrained using the LINCS algorithm, and long-range electrostatic interactions were computed with the Particle Mesh Ewald (PME) method (Yu et al., 2020). After equilibration, each system was subjected to a 100-nanosecond production run under constant pressure and temperature conditions, during which atomic trajectories were collected for subsequent structural and dynamic analyses.

The binding free energy of the protein–ligand complex was estimated using the *gmx_MMPBSA* tool (v1.6.3), which integrates GROMACS with AmberTools for end-state free energy analysis (Valdés-Tresanco et al., 2021). The total binding energy was decomposed into van der Waals, electrostatic, and solvation (polar and nonpolar) components. The Generalized Born (GB) implicit solvent model with igb = 5 (GB-Neck2) was applied for polar solvation, while the LCPO method was used for nonpolar contributions. Entropy terms were excluded, consistent with common practice in comparative MM/GBSA studies (Genheden and Ryde, 2012). A total of 1,001 snapshots were extracted uniformly from the 100-ns production trajectory, and calculations were performed for the complex, receptor, and ligand to obtain the final binding energy.

### 2.10 Immune simulation

The immune response profile of individual epitopes as well as the designed multi-epitope pan-coronavirus vaccine construct was evaluated *in silico* using the C-ImmSim server (http://150.146.2.1/C-IMMSIM/index.php), an agent-based simulation platform that predicts mammalian immune kinetics through position-specific scoring matrix (PSSM) and machine-learning algorithms (Rapin et al., 2010). The simulator models the interactions among immune cells, cytokines, and antigens over discrete time steps, allowing visualization of both primary and secondary immune responses.

To align with the study objectives and reported results, two simulation schemes were applied. First, a baseline single-dose epitope-level simulation was conducted for the selected CTL and HTL epitopes, using one injection at time step 1 to assess the intrinsic immunostimulatory potential of the epitopes when evaluated in isolation. Second, the multi-epitope vaccine construct was evaluated under a prime–boost regimen consisting of three injections at time steps 1, 84, and 168, corresponding to 0, 4, and 8 weeks in real time, with each injection containing 1,000 vaccine molecules.

Default parameters were used for random seed generation, simulation volume, and baseline host immune cell populations. The HLA alleles specified in the simulator were matched to those considered in the population coverage analysis to maintain representational diversity across global haplotypes. Simulation outputs included the kinetics of B-cell, HTL, and CTL populations, antibody titers (IgM, IgG, IgG1, IgG2, and immune complexes), cytokine profiles (including IL-2 and IFN-γ), and memory cell formation. The outputs were interpreted to compare response magnitude, persistence, and coordination between the baseline single-dose epitope simulation and the prime–boost multi-epitope construct simulation, with sustained IgG responses and persistent memory B and T cells taken as indicators of durable immunological memory and predicted vaccine efficacy (Turner et al., 2021; McLean et al., 2023).

## 3 Results and discussion

### 3.1 Protein sequence collection and multiple sequence alignment

Protein sequences were retrieved from the NCBI database. A total of 22 S1 receptor-binding domain (RBD) protein sequences were collected, comprising seven sequences each for SARS-CoV and MERS-CoV and eight sequences for SARS-CoV-2. Sequence selection prioritized geographic diversity to provide representative coverage across regions and hosts, and the MERS-CoV dataset included both human and non-human isolates as listed in Supplementary Table S1. The inclusion of animal-derived MERS-CoV sequences was justified because dromedary camels are the primary reservoir for zoonotic transmission and camel-derived MERS-CoV strains show very high genetic similarity to human isolates, including reports of near-identical camel–human transmission pairs and very high genome-level similarity, supporting their use as biologically relevant surrogates for human infection (Azhar et al., 2014; Sabir et al., 2016; Paden et al., 2017). The SARS-CoV-2 sequences in this panel predominantly represent ancestral or pre-Omicron diversity, based on the available lineage annotations for the selected accessions, and Omicron-era lineages were not included in the epitope discovery dataset.

**FIGURE 1:**
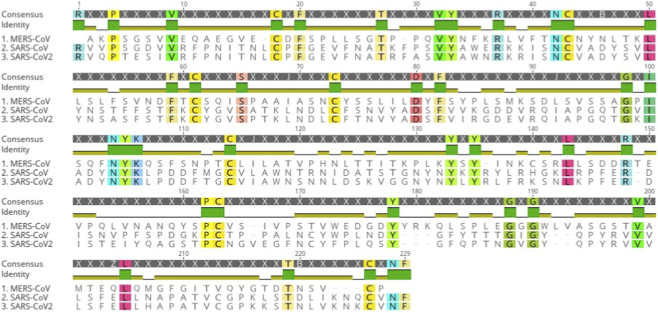
Multiple sequence alignment of the S1 receptor-binding domain (RBD) from SARS-CoV, MERS-CoV, and SARS-CoV-2 generated using the full 22-sequence dataset (7 SARS-CoV, 7 MERS-CoV, and 8 SARS-CoV-2), including non-human MERS-CoV isolates. For clarity, representative sequences are shown (SARS-CoV, GenBank ID: ADC35483; MERS-CoV, GenBank ID: AGN70929; SARS-CoV-2, GenBank ID: QHR63270). Conserved positions and segments defined from the complete alignment are indicated by consistent residue patterns and shading and were used for downstream feature extraction and epitope prediction.

Multiple sequence alignment of the complete 22-sequence dataset was performed to evaluate sequence conservation and to define conserved positions and segments within each viral group. Conserved segments identified from the full alignment were subsequently carried forward for downstream feature extraction and epitope prediction. For clarity, Figure 1 presents representative sequences (one per virus group) to illustrate the conserved segments derived from the complete MSA. The observed conservation supports the functional relevance of these segments and motivates their consideration as candidate targets for broadly protective coronavirus vaccine design (Cohen et al., 2022; Wec et al., 2020).

### 3.2 Prediction of B- and T-Cell epitopes

Linear B-cell epitope prediction identified several immunogenic regions within the S1 RBD of SARS-CoV, MERS-CoV, and SARS-CoV-2. Epitopes ranging from 5 to 30 amino acids were considered suitable for further evaluation (Yang et al., 2025). Based on antigenicity and structural criteria, five epitopes were selected for SARS-CoV, two for MERS-CoV, and three for SARS-CoV-2, representing promising candidates for broad-spectrum betacoronavirus vaccine design.

To complement the B-cell analysis, a comprehensive panel of high-frequency HLA alleles was incorporated for T-cell epitope prediction to ensure broad population coverage (Table 1). For MHC class I, twenty-one alleles were included, representing the most prevalent HLA-A, -B, and -C supertypes globally (e.g., A02:01, A24:02, B07:02, and C07:02). For MHC class II, sixteen DRB1 alleles were used, encompassing major variants such as DRB103:01, DRB104:05, DRB107:01, and DRB115:01, which collectively provide extensive ethnic and geographical representation. The inclusion of these alleles, as recommended by previous studies (Jin et al., 2022; Wolday et al., 2023; Samad et al., 2020), enhances the predictive relevance of the identified epitopes by maximizing immunogenetic diversity and ensuring their potential applicability across global human populations.

**TABLE 1:** List of high-frequency HLA alleles used for MHC class I and class II T-cell epitope prediction. The selected alleles represent globally prevalent variants to ensure broad population coverage and immunogeneticdiversity.

	Selected high-frequency HLA alleles
MHC class I	A*01:01, A*02:01, A*02:03, A*02:07, A*11:01, A*24:02, A*24:07, A*29:01, A*30:01, A*34:01, B*07:02, B*15:01, B*15:02, B*40:01, B*44:03, B*46:01, B*51:01, C*01:02, C*07:02, C*08:01, C*16:01
MHC class II	DRB1*03:01, DRB1*04:05, DRB1*04:07, DRB1*07:01, DRB1*08:01, DRB1*08:02, DRB1*08:04, DRB1*09:01, DRB1*11:01, DRB1*12:02, DRB1*13:02, DRB1*14:05, DRB1*14:06, DRB1*15:01, DRB1*15:02, DRB1*15:03

T-cell epitope prediction was subsequently conducted to identify peptides capable of binding to multiple HLA alleles. Epitopes selected for further analysis were those predicted to interact with at least three overlapping HLA alleles, ensuring broad coverage across diverse genetic backgrounds (Suar et al., 2020). For MHC class I, selected epitopes exhibited percentile ranks between ≤0.5% (strong binders) and ≤2% (weak binders), whereas for MHC class II, the percentile rank range was ≤2% (strong binders) to ≤10% (weak binders) (Shaikh et al., 2024).

### 3.3 Immunogenicity evaluation of predicted B-Cell epitopes and T-Cell epitopes

The comprehensive immunogenicity evaluation of predicted linear B-cell, CTL (MHC class I), and HTL (MHC class II) epitopes demonstrated that all selected peptides exhibited favorable immune-relevant and safety characteristics (Table 2). For B-cell epitopes, all peptides from SARS-CoV, MERS-CoV, and SARS-CoV-2 showed antigenicity values above the established viral threshold, indicating their potential to trigger humoral immune responses. The SARS-CoV-2 epitope displayed the highest antigenicity (0.9322), followed by SARS-CoV (0.8940) and MERS-CoV (0.6850), suggesting strong immunogenic capacity across all three viruses.

**TABLE 2:** Immunogenicity, allergenicity, toxicity, and homology profiles of the selected linear B-cell, cytotoxic T-lymphocyte (CTL; MHC class I), and helper T-lymphocyte (HTL; MHC class II) epitopes predicted from SARS-CoV, MERS-CoV, and SARS-CoV-2.

	B-Cell epitopes				
	Epitope	Antigenicity	Allergenicity	Toxicity	Homology
SARS-CoV	VVKGDDVRQIAPGQTGVIADYNYKLPD	Probable antigen 0.8940	Probable non-allergen	Non-toxic	Non-homologous
MERS-CoV	YPLSMKSDLSVSSAGPISQFNYKQSFSN	Probable antigen 0.6850	Probable non-allergen	Non-toxic	Non-homologous
SARS-CoV 2	IRGDEVRQIAPGQTGKIADYNYKLPD	Probable antigen 0.9322	Probable non-allergen	Non-toxic	Non-homologous

	Cytotoxic T-lymphocyte (CTL; MHC class I) epitopes				
	Epitope	Antigenicity	Allergenicity	Toxicity	Homology
SARS-CoV	YQPYRVVVL	Probable antigen 0.5964	Probable non-allergen	Non-toxic	Non-homologous
MERS-CoV	SQFNYKQSF	Probable antigen 1.1913	Probable non-allergen	Non-toxic	Non-homologous
SARS-CoV 2	GQTGKIADY	Probable antigen 1.4019	Probable non-allergen	Non-toxic	Non-homologous

	T-lymphocyte (HTL; MHC class II) epitopes				
	Epitope	Antigenicity	Allergenicity	Toxicity	Homology
SARS-CoV	DYGFYTTTGIGYQPY	Probable antigen 1.4066	Probable non-allergen	Non-toxic	Non-homologous
MERS-CoV	DGDYYRKQLSPLEGG	Probable antigen 0.7241	Probable non-allergen	Non-toxic	Non-homologous
SARS-CoV 2	RKSNLKPFERDISTE	Probable antigen 0.4847	Probable non-allergen	Non-toxic	Non-homologous

Similarly, the CTL epitopes exhibited robust antigenicity, suggesting their ability to activate cytotoxic immune responses. The SARS-CoV-2 epitope GQTGKIADY recorded the highest score (1.4019), followed by MERS-CoV (SQFNYKQSF, 1.1913) and SARS-CoV (YQPYRVVVL, 0.5964), indicating consistently strong immunogenic profiles across conserved viral regions. For HTL epitopes, all candidates demonstrated antigenicity above the viral threshold (González-Cruz et al., 2025), highlighting their potential to elicit helper T-cell responses. The SARS-CoV epitope DYGFYTTTGIGYQPY showed the highest antigenicity (1.4066), followed by MERS-CoV (DGDYYRKQLSPLEGG, 0.7241) and SARS-CoV-2 (RKSNLKPFERDISTE, 0.4847).

Across all epitope classes, the predicted peptides were identified as non-allergenic, non-toxic, and non-homologous to human proteins, indicating excellent safety and minimal risk of autoimmune cross-reactivity. These findings confirm the identified B-, CTL-, and HTL-epitopes as strong, safe, and immunogenic candidates for incorporation into a multi-epitope pan-betacoronavirus vaccine construct aimed at eliciting broad and durable immune protection.

### 3.4 Population coverage

Human leukocyte antigen (HLA) polymorphism influences inter-individual variability in epitope presentation and, consequently, immune responsiveness. Therefore, selecting epitopes with broad HLA representation is important for maximizing the potential applicability of multi-epitope vaccine constructs across populations. The six selected MHC class I and class II epitopes were assessed using the IEDB population coverage tool, which estimates the proportion of individuals predicted to carry at least one HLA allele capable of presenting at least one of the evaluated epitopes. The combined epitope set yielded an estimated global population coverage of 93.28%, with an average hit of 3.28 and a predicted coverage for 90% of the population (PC_90_) of 1.24. These results indicate extensive predicted HLA representation across diverse human populations and support the construct’s potential for broad population-level applicability in terms of predicted HLA presentation (Bui et al., 2006).

### 3.5 Multi-epitope pan-betacoronavirus vaccine construct design and structural modelling

The proposed multi-epitope pan-betacoronavirus vaccine was designed to elicit broad humoral and cellular immune responses by combining B-cell, CTL, and HTL epitopes derived from the receptor-binding domains of SARS-CoV, MERS-CoV, and SARS-CoV-2. The construct incorporates human β-defensin 3 as an adjuvant to enhance immunogenicity through activation of antigen-presenting cells (Ferris et al., 2012), and the PADRE sequence to promote T-helper responses and overcome HLA polymorphism (Hung et al., 2007). The final assembled sequence comprised 285 amino acids.

A rigid EAAAK linker was used to separate the adjuvant from the epitope region, thereby maintaining structural stability, whereas AAY and GPGPG linkers were incorporated between CTL and HTL epitopes, respectively, to facilitate proper antigen processing and MHC presentation (Choudhury et al., 2024). The GGGGS-based flexible linkers connecting B-cell epitopes provided conformational flexibility for optimal antibody recognition. The vaccine construct was rationally assembled by integrating conserved epitopes from multiple coronaviruses with suitable adjuvant and linker sequences, ensuring balanced activation of humoral and cell-mediated immunity as well as broad population coverage against diverse betacoronavirus strains.

**FIGURE 2:**
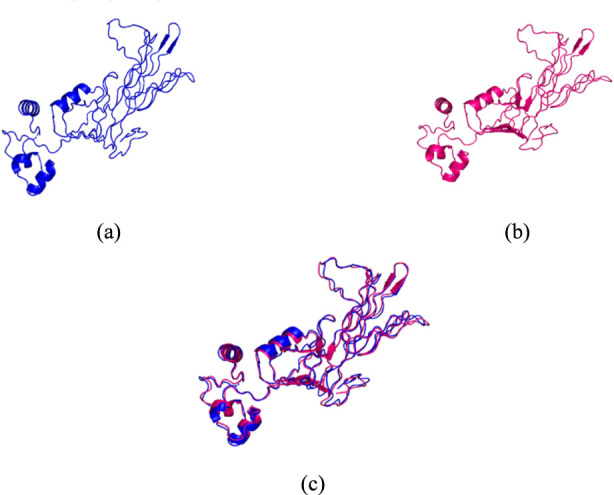
Visualization of the vaccine model: **(a)** before refinement, **(b)** after refinement, and **(c)** structural superimposition of the pre-refined (blue) and post-refined (pink) models. Local conformational adjustments, particularly in the loop and helical regions, indicate refinement-induced changes in atomic positioning and improved structural stability following refinement.

The vaccine model with the highest C-score (−3.60) among all predicted structures was selected as the initial construct. This value lies within the acceptable confidence interval (−5 to 2), where higher C-scores indicate greater reliability and closer resemblance to the native protein conformation (Al-Zyoud and Haddad, 2020). To enhance its three-dimensional accuracy, the model underwent a two-step refinement process (Figure 2), an approach validated in previous studies demonstrating its effectiveness in producing models that more closely approximate experimentally resolved structures (Kathwate, 2020). The refined model achieved a TM-score of 0.9850 and an RMSD of 0.771 Å. In general, TM-scores >0.5 are commonly interpreted as indicating correct overall fold, whereas values closer to 1.0 denote near-identical global topology. Similarly, RMSD values below approximately 2 Å are typically considered indicative of high structural similarity in comparative model assessment, with lower values reflecting tighter structural superposition. Therefore, the TM-score approaching 1.0 together with the sub-angstrom RMSD supports very high similarity between the refined and reference structures and is consistent with precise refinement (Olechnovi et al., 2018; Heo and Feig, 2018).

Structural validation using a Ramachandran plot (Supplementary Figure S1) indicated improved stereochemical quality after refinement. Before refinement, 85.1% of residues were in the most favoured regions, 13.4% in additional allowed regions, 0.5% in generously allowed regions, and 1.0% in disallowed regions. After refinement, 88.6% of residues were in the most favoured regions, 10.9% in additional allowed regions, 0.5% in generously allowed regions, and no residues were observed in disallowed regions, indicating elimination of backbone outliers and improved stereochemical plausibility.

### 3.6 Molecular docking of the vaccine construct

The refined multi-epitope vaccine construct was docked with the B-cell receptor (BCR) to assess its potential for humoral immune recognition. Docking with MHC class I and II molecules was not conducted because these molecules physiologically bind short peptide fragments generated through proteolytic processing within antigen-presenting cells (APCs) such as dendritic cells, macrophages, and B cells (Roche and Furuta., 2015). Docking the intact vaccine construct with MHC molecules would therefore not reflect the natural antigen presentation mechanism. In contrast, BCR docking mimics the early stage of immune recognition, in which native antigens are directly bound by surface immunoglobulins on B cells. This interaction provides meaningful insights into the construct’s potential to enable structurally plausible Fab/BCR engagement at an antibody-like interface (Wen et al., 2019). Accordingly, the docking analysis is interpreted as a complementary predictive step that provides a structural rationale for potential B-cell recognition and interface plausibility.

**FIGURE 3:**
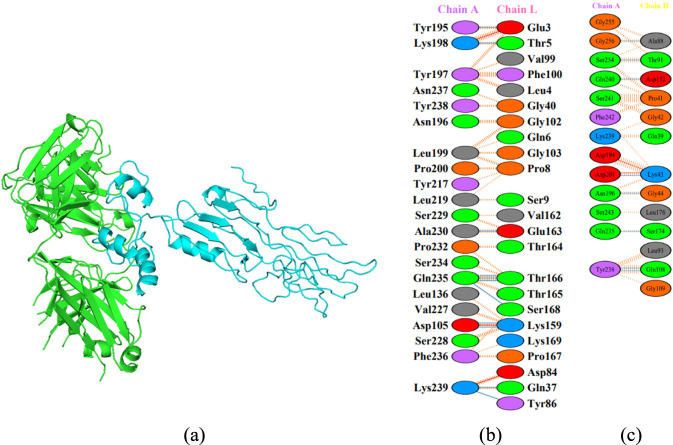
Three-dimensional molecular docking of the refined pan-betacoronavirus multi-epitope vaccine construct with the BCR Fab model. **(a)** The vaccine construct (light blue) forms an extensive predicted interface with the BCR (green). The selected complex was obtained from a highly populated ClusPro cluster (Cluster 1; 49 members) and exhibited a Lowest Energy score of −865.9. **(b)** Interaction map between the vaccine construct (Chain A) and the BCR light chain (L), showing three predicted salt-bridge contacts (Lys198-Glu3, Asp105-Lys159, and Lys239-Asp84) and four intermolecular hydrogen bonds. **(c)** Interaction map between the vaccine construct and the BCR heavy chain (H), showing a predicted salt-bridge contact (Asp194–Lys43) and nine predicted hydrogen bonds, involving residues such as Gln235, Asn196, and Tyr238. Blue solid lines represent hydrogen bonds, red solid lines represent salt bridges, and orange dashed lines indicate non-bonded hydrophobic contacts. These interactions are shown to illustrate interface complementarity and pose plausibility under the docking scoring function.

Molecular docking of the post-refinement multi-epitope vaccine construct with the BCR yielded a ClusPro Lowest Energy score of −865.9 for the selected vaccine–BCR complex, selected from a highly populated cluster (Cluster 1; 49 members) (Supplementary Table S2). ClusPro rankings reflect relative pose quality and interface complementarity rather than absolute binding affinity. Accordingly, more negative ClusPro Lowest Energy scores indicate more favorable predicted binding poses under the ClusPro scoring function and are used to rank modeled complexes comparatively (Kozakov et al., 2017). Visualization of the vaccine–BCR complex (Figure 3a) revealed extensive interfacial complementarity, involving multiple hydrogen bonds, hydrophobic interactions, and electrostatic contacts, which are commonly associated with stable antibody–antigen interfaces (Dar et al., 2019).

The interaction maps further illustrate the specific residue-level contacts between the vaccine construct (Chain A) and the B-cell receptor (BCR), encompassing the light chain (Chain L) (Figure 3b) and the heavy chain (Chain H) (Figure 3c). A detailed analysis of these interfaces identifies a robust network of 13 intermolecular hydrogen bonds and 4 primary salt-bridge pairs. On the light chain, Lys198, Asp105, and Lys239 from the vaccine construct form critical salt bridges with BCR residues Glu3, Lys159, and Asp84, respectively. On the heavy chain, Asp194 establishes a strong ionic anchor through a salt bridge with Lys43. The hydrogen-bonding network is primarily anchored by Gln235, which acts as a dual-chain stabilizer by engaging both Thr166 (L) and Ser174 (H). Additionally, residues Asn196, Asp201, Tyr238, Ser254, and Gly256 form specific polar interactions with the heavy chain. Furthermore, the involvement of aromatic residues—including Tyr195, Tyr197, Tyr217, Tyr238, and Phe236—provides significant hydrophobic stabilization and enhances surface complementarity through van der Waals interactions. The main binding interface spans residues Tyr195 to Gly256 on the vaccine construct, targeting highly accessible loops and β-sheet regions on the BCR paratope that are essential for high-affinity antibody recognition and immune signaling. The details of the interacting residues are provided in Supplementary Table S3.

Overall, the docking-derived contact pattern supports a multivalent, spatially distributed antibody-like interface and indicates a structurally feasible engagement mode under the ClusPro scoring function. Because docking was performed against a generic, non–coronavirus-specific Fab/BCR model within a static structural framework, these results should be interpreted strictly as structure-based plausibility rather than functional evidence: they do not establish antigen specificity, BCR repertoire prevalence, *in vivo* receptor cross-linking, or downstream signaling outcomes. Accordingly, mechanistic statements about BCR cross-linking and subsequent intracellular signaling, including Lyn and Syk activation, as well as downstream proliferation, differentiation, and antibody production, should be treated as immunological context rather than direct outputs of the docking analysis (Wen et al., 2019).

Within these constraints, the favorable Fab-like pose has implications for pan-betacoronavirus vaccine design at the level of structural feasibility. Because the construct incorporates epitopes from conserved RBD regions shared among SARS-CoV, MERS-CoV, and SARS-CoV-2, the docking results support the plausibility that conserved RBD features can adopt antibody-accessible conformations compatible with Fab-like engagement. However, breadth-related claims such as cross-reactivity, cross-neutralization, and protection across lineages remain hypotheses and require coronavirus-specific validation, including epitope mapping, binding kinetics using relevant antibodies or BCR repertoires, and neutralization assays.

In this context, references to broadly neutralizing antibodies such as S309 and ADG-2 are presented as conceptual parallels supporting the strategy of targeting conserved RBD epitopes, not as evidence of structural or mechanistic equivalence. S309 recognizes a conserved, glycan-containing site outside the receptor-binding motif and can mediate cross-reactive neutralization of SARS-CoV-2, whereas ADG-2 targets a highly conserved RBD epitope overlapping the ACE2-binding site and shows broad neutralization across sarbecoviruses (Pinto et al., 2020). Unlike these bnAbs, which are supported by epitope-resolved structural and functional datasets, the present docking analysis is intended to evaluate interface plausibility and pose quality for an antibody-like interaction with the designed construct.

From a translational perspective, a construct exhibiting favorable antibody-like interface complementarity to a generic Fab/BCR model may serve as an initial structural feasibility indicator for breadth-oriented vaccine design. Nevertheless, claims regarding reformulation mitigation, cross-neutralization breadth, and protection against emergent lineages should be framed as hypotheses that require validation using betacoronavirus-specific antibodies/BCR repertoires and functional immunogenicity and neutralization assays.

### 3.7 Molecular dynamics simulation and system stability analysis

**FIGURE 4:**
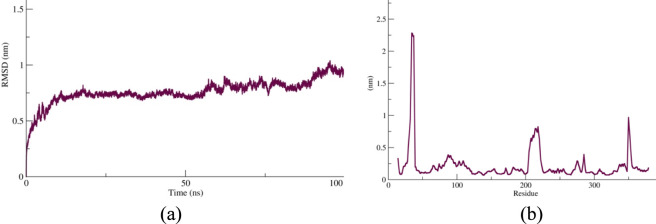
**(a)** RMSD profile of the vaccine construct demonstrating rapid equilibration within the first 10 ns and sustained structural stability throughout the 100-ns molecular dynamics simulation. **(b)** RMSF profile illustrating predominantly low residue fluctuations (<0.5 nm), with higher flexibility observed around residues 20–40, 190–220, and ∼330, corresponding to loop and surface-exposed regions. The overall pattern indicates a stable backbone with localized dynamic regions that may facilitate receptor interaction while maintaining global structural integrity.

The RMSD and RMSF analyses collectively confirmed the dynamic stability of the protein–ligand complex during the 100-ns molecular dynamics simulation (Figures 4a,b). The RMSD plot showed a rapid increase within the first 10 ns, stabilizing around 0.6 nm, which reflects rapid equilibration of the protein backbone in the solvent environment—an expected trend in well-equilibrated molecular systems (Geisbrecht et al., 2002; Hollingsworth and Dror, 2018). After approximately 25 ns, only minor fluctuations (0.5–0.9 nm) were observed, and a slight rise toward ∼1.1 nm after 80 ns indicated limited domain flexibility rather than unfolding, consistent with the conformational breathing typically reported for stable protein–ligand complexes (Sastry et al., 2013).

Complementary to this, the RMSF profile demonstrated that most residues displayed low atomic fluctuations (<0.5 nm), indicating a stable backbone structure with limited flexibility. Only a few peaks—particularly around residues 20–40, 190–220, and approximately residue 330—showed elevated mobility (up to ∼2.2 nm), corresponding to surface-exposed loop regions that contribute to molecular recognition and receptor engagement. Such localized flexibility, combined with overall structural rigidity, is characteristic of dynamic yet conformationally stable proteins (Lindorff-Larsen et al., 2011). The minimal fluctuations observed in core residues suggest preserved structural integrity and stable intramolecular interactions throughout the simulation. These findings are consistent with earlier molecular dynamics studies linking low RMSD and RMSF deviations to enhanced conformational stability and binding persistence in protein systems (Ahmad and Patel, 2025), confirming that the vaccine construct maintained a compact and well-equilibrated structure during the entire simulation period.

**FIGURE 5:**
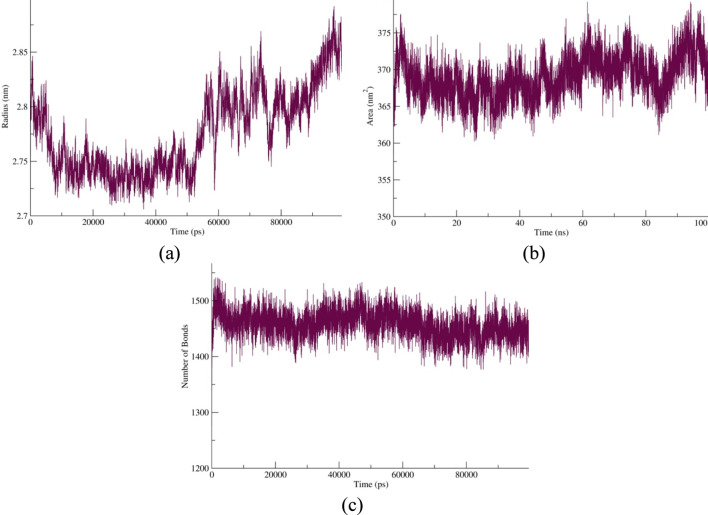
**(a)** Radius of gyration (Rg) showing sustained structural compactness of the protein–ligand complex. **(b)** Solvent-accessible surface area (SASA) indicating stable solvation and surface exposure. **(c)** Hydrogen bond profile demonstrating consistent intermolecular interactions and structural stability throughout the 100-ns simulation.

The radius of gyration (Figure 5a) demonstrated an early decrease from 2.85 nm to 2.73 nm within the first 20 ns, reflecting initial structural compaction as the system reached equilibrium. Such behavior is characteristic of stable protein–ligand systems, where solvent relaxation and ligand binding promote tighter packing of the backbone (Geisbrecht et al., 2002). Thereafter, the Rg values remained nearly constant between 2.74 and 2.80 nm, suggesting that the complex maintained a compact and equilibrated conformation with only minor conformational breathing toward the end of the trajectory. This stability aligns with previous simulation reports describing comparable Rg plateaus in thermodynamically stable macromolecular complexes (Torrens-Fontanals et al., 2020).

The solvent-accessible surface area (Figure 5b) exhibited minor oscillations between 360 nm^2^ and 375 nm^2^, averaging around 367 nm^2^, consistent with stable solvation and a well-folded tertiary structure. This steady SASA pattern supports the notion that ligand association reduces solvent exposure of hydrophobic residues, contributing to overall conformational stability (Wimbarti et al., 2025). Similarly, the hydrogen bond profile (Figure 5c) remained steady throughout the 100-ns trajectory, averaging approximately 1,450 bonds with minor fluctuations, indicative of a persistent intra- and intermolecular hydrogen-bonding network. The maintenance of these bonds reflects strong structural cohesion and continuous solvation, as also reported in comparable MD studies of stable protein–ligand complexes (Tallei et al., 2025). These parameters confirm that the protein–ligand complex remained compact, well-folded, and dynamically stable under simulated physiological conditions.

The MM/GBSA analysis (Table 3) showed a favorable binding affinity (ΔG_bind_ = −7.46 kcal/mol), indicating a spontaneous and thermodynamically stable protein–ligand interaction. The dominant stabilizing contribution arose from van der Waals forces (−10.62 kcal/mol), highlighting the importance of hydrophobic and dispersion interactions in complex stabilization (Tallei et al., 2025; Ferenczy and Kellermayer, 2022). Electrostatic interactions were modest (−1.01 kcal/mol), while the unfavorable polar solvation energy (+5.58 kcal/mol) was counterbalanced by the stabilizing nonpolar solvation term (−1.40 kcal/mol). Overall, the net binding energy confirmed that favorable hydrophobic and van der Waals contributions outweighed solvation penalties, in agreement with earlier MM/GBSA studies reporting similar energetic patterns in stable biomolecular complexes (Mandal and Mandal, 2024).

**TABLE 3:** MM/GBSA binding-free energy components of the protein–ligand complex showing contributions from van der Waals, electrostatic, and solvation energies. The negative total ΔG value indicates a favorable and spontaneous binding interaction.

Energy component		Value (kcal/mol)
E_vdW_	van der Waals	−10.62
E_ele_	Electrostatic	−1.01
E_polar_	Polar solvation	5.58
E_nonpolar_	Nonpolar solvation	−1.4
G_gas_	Gas-phase	−11.63
G_solv_	Solvation free	4.18
ΔG_bind_	Total binding-free	−7.46

### 3.8 Immune simulation of predicted CTL and HTL epitopes

**FIGURE 6:**
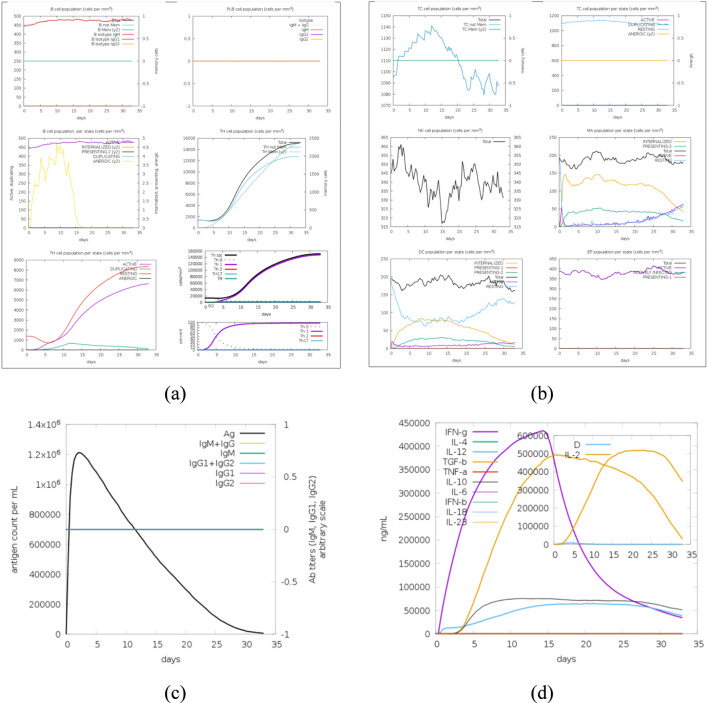
Baseline single-dose, epitope-level *in silico* immune response simulation of the selected CTL (YQPYRVVVL, SQFNYKQSF, GQTGKIADY) and HTL (DYGFYTTTGIGYQPY, DGDYYRKQLSPLEGG, RKSNLKPFERDISTE) epitopes using the C-IMMSIM server. **(a)** Immune cell population dynamics showing initial antigen uptake and moderate activation of B and T lymphocytes, followed by limited expansion and rapid contraction typical of isolated epitope exposure. **(b)** Immunoglobulin kinetics demonstrating a transient IgM peak with minimal IgG class switching, indicating weak and short-lived humoral activation. **(c)** Cytokine secretion profiles illustrating brief IL-2 and IFN-γ spikes consistent with transient T-cell priming, followed by rapid decline, suggesting limited immune persistence. **(d)** Memory cell profiles showing a modest rise and quick decay in memory B and T lymphocytes, consistent with insufficient long-term memory formation in a single-dose epitope-only setting.

To establish a baseline, single-dose epitope-level response, the *in silico* immune simulation was performed using the selected CTL epitopes (YQPYRVVVL, SQFNYKQSF, GQTGKIADY) and HTL epitopes (DYGFYTTTGIGYQPY, DGDYYRKQLSPLEGG, RKSNLKPFERDISTE). This single-exposure simulation demonstrated a modest activation of both humoral and cellular immune components (Figures 6a–d). The lymphocyte population dynamics (Figure 6a) showed an initial phase of antigen uptake and activation, followed by a limited expansion of B and T cells that declined rapidly, indicating transient immune stimulation. The antibody kinetics (Figure 6b) revealed a brief IgM response with minimal IgG class switching, reflecting a weak humoral response. Correspondingly, cytokine secretion profiles (Figure 6c) displayed early but short-lived IL-2 and IFN-γ peaks, signifying limited T-cell priming and a short Th1-biased response. The memory cell profile (Figure 6d) showed only a modest rise in memory B and T lymphocytes, which subsided after antigen clearance, suggesting insufficient long-term memory formation.

Within the simulator’s intrinsic analyses, Parker B-propensity plots identified multiple antigenic stretches within the input peptides, including GQTGKIADY in SARS-CoV-2, DGDYYR in the MERS-CoV HTL peptide, and additional regions within the SARS-CoV-2 HTL epitope, supporting their inherent B-cell–facing antigenicity. Although these individual epitopes exhibited theoretical immunogenic potential, the overall immune kinetics in this baseline single-dose simulation indicated that they are insufficient to elicit sustained or protective immune responses. This finding underscores the rationale for integrating multiple conserved epitopes into a single multi-epitope construct and evaluating it under a prime–boost regimen (Section 3.9) to achieve synergistic activation of humoral and cellular immunity, enhance cross-reactive recognition across conserved RBD regions of SARS-CoV, MERS-CoV, and SARS-CoV-2, and promote broader, more durable pan-coronavirus protection.

### 3.9 Immune simulation of the multi-epitope pan-betacoronavirus vaccine construct

**FIGURE 7:**
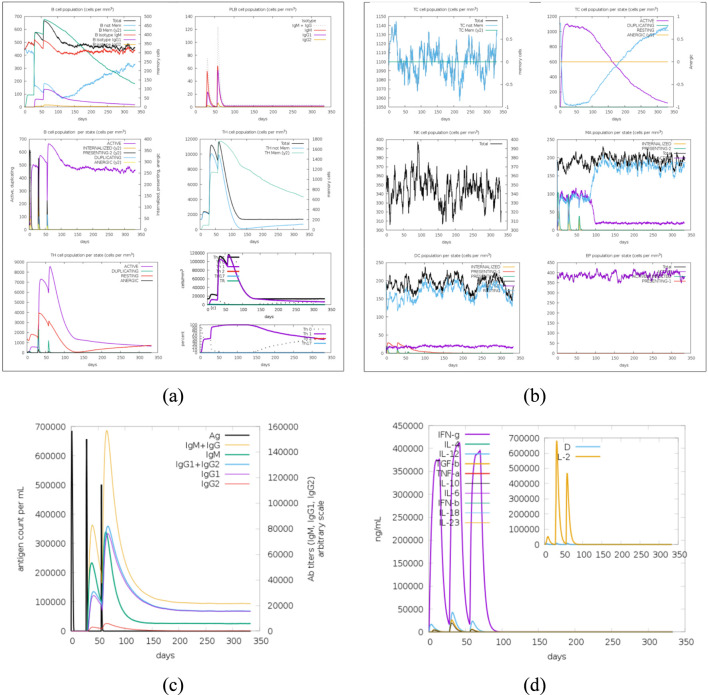
Prime–boost *in silico* immune response simulation of the multi-epitope pan-coronavirus vaccine construct using the C-IMMSIM server. **(a)** Lymphocyte population profiles showing strong activation of antigen-presenting cells followed by robust expansion of B cells, cytotoxic T lymphocytes (CTLs), and helper T lymphocytes (HTLs), indicating coordinated engagement of humoral and cellular immunity. **(b)** Antibody titers and antigen levels across successive exposures, demonstrating an initial IgM peak after priming and amplified IgG-dominated responses after booster doses, consistent with effective class switching and establishment of long-term humoral memory. **(c)** Cytokine secretion kinetics showing pronounced IL-2 and IFN-γ peaks consistent with Th1 polarization and T-cell priming, with sustained cytokine production reflecting prolonged immune stimulation. **(d)** Memory cell dynamics illustrating persistently elevated memory B and T lymphocytes following boosting, supporting durable immune memory and the construct’s potential for broad, cross-reactive protection.

To complement the baseline single-dose epitope-level simulation (Section 3.8), the multi-epitope vaccine construct was evaluated under a prime–boost regimen. The *in silico* immune simulation demonstrated a robust and long-lasting adaptive immune response following administration of the multi-epitope construct (Figures 7a–d). The lymphocyte population dynamics (Figure 7a) showed strong and sustained activation of antigen-presenting cells, followed by rapid proliferation of B cells, CTLs, and HTLs, indicating efficient engagement of both humoral and cellular immune arms. The antibody response (Figure 7b) exhibited a typical biphasic pattern, an early IgM surge during the primary response and a pronounced IgG peak upon secondary and tertiary exposures, signifying effective class switching and formation of long-term humoral memory.

The cytokine secretion profile (Figure 7c) revealed marked peaks of IL-2 and IFN-γ, consistent with strong Th1 polarization, T-cell priming, and cytotoxic activity critical for antiviral defense. Sustained cytokine production over time also reflected prolonged immune stimulation. The memory cell analysis (Figure 7d) displayed persistently elevated levels of both memory B and T cells, supporting the development of durable immunological memory with potential for rapid recall upon re-exposure.

These results suggest that, within the C-ImmSim immune simulation framework using default parameters, the multi-epitope construct is predicted to elicit a more balanced and persistent humoral and cellular response profile than the corresponding single-epitope simulations. The integration of conserved epitopes from SARS-CoV, MERS-CoV, and SARS-CoV-2, together with broad HLA representation, supports its potential as an *in silico* pan-betacoronavirus vaccine candidate, while recognizing that the breadth and durability of protection require experimental validation.

### 3.10 Comparative analysis of immune simulation between single-epitope and multi-epitope constructs

The immune simulation of the multi-epitope vaccine construct under a prime–boost regimen predicted a robust and well-orchestrated response within the C-ImmSim framework, indicative of strong immunogenic potential and model-inferred durability. Importantly, these immune simulations primarily model cellular immune dynamics driven by the T-cell epitope inputs and the parameterization of the computational framework (Cheng et al., 2022). Following priming, the model predicted rapid activation of antigen-presenting cells was accompanied by expansion of both B- and T-lymphocyte populations. Subsequent booster exposures were predicted to markedly amplified responses, reflected by sharp increases in plasma cells, memory B cells, and both HTL and CTL populations.

The antibody profile was predicted to show an early IgM surge followed by pronounced IgG1 and IgG2 peaks after boosting, consistent with simulated class switching and establishment of long-term humoral immunity. Cytokine profiling predicted elevated IL-2 and IFN-γ levels, supporting a Th1-skewed response relevant to antiviral defense and cytotoxic activity, and consistent in the model with enhanced CD8^+^ T-cell proliferation and memory formation. Persistently increased memory B- and T-cell populations throughout the simulation further suggested, within the model, the construct’s capacity to sustain long-term immune memory and enable rapid recall upon re-exposure.

In contrast, the baseline single-dose, epitope-level simulations were predicted to produce weaker and short-lived immune responses characterized by transient IgM peaks, limited cytokine release, and minimal memory cell development. These findings suggest, within the simulation setting, that isolated CTL or HTL peptides, despite predicted immunogenicity, are insufficient to drive sustained humoral or cellular immunity when evaluated individually under a single-exposure setting. This limitation is also consistent with the theoretical HLA restriction of single epitopes, which bind only a subset of alleles and therefore may confer protection in a narrower segment of the population.

By integrating multiple conserved RBD epitopes from SARS-CoV, MERS-CoV, and SARS-CoV-2 into a single construct, the multi-epitope design was predicted to provide broader HLA allele coverage (93.28%) as indicated by the population coverage analysis. This immunogenetic inclusivity is expected to increase the likelihood of eliciting effective immune responses across diverse ethnic and geographic populations. Overall, the comparison indicates, within the C-ImmSim simulations using default parameters, that the multi-epitope strategy enhances immunogenicity through synergistic activation of B- and T-cell pathways and supports immune memory, thereby strengthening its potential as an *in silico* pan-betacoronavirus vaccine candidate for broad and long-lasting protection pending experimental validation against divergent betacoronavirus strains.

### 3.11 Prior broad-spectrum betacoronavirus vaccine efforts

Recent broad-spectrum coronavirus vaccine efforts support the rationale for targeting conserved epitopes and applying integrated computational pipelines, particularly because strain-matched vaccines can be limited by antigenic evolution and waning immunity (Cankat et al., 2023). In this context, our construct differs from many breadth-oriented candidates that primarily address SARS-CoV-2 variant breadth or focus on a single subgenus, because it integrates conserved RBD-derived epitopes spanning both sarbecoviruses (SARS-CoV and SARS-CoV-2) and merbecoviruses (MERS-CoV), thereby representing a cross-subgenus betacoronavirus epitope design within one multi-epitope construct. Large-scale epitope mapping and conservation analyses further support the biological plausibility of breadth-oriented epitope selection by demonstrating that conserved T-cell epitope regions can be cross-reactively recognized across multiple betacoronavirus subgenera, indicating that cross-subgenus T-cell reactivity is feasible and not restricted to within-lineage responses (Neto et al., 2025).

While most immunoinformatics multi-epitope studies have concentrated on SARS-CoV-2 strain or variant coverage, prior RBD-focused epitope screening across MERS-CoV, SARS-CoV, and SARS-CoV-2 provides methodological precedent for a tri-virus RBD-centered strategy and highlights the relative scarcity of epitope-string constructs explicitly integrating epitopes across these three pathogenic betacoronaviruses (Khan et al., 2022). Experimental breadth-oriented strategies are also emerging *in vivo*; for example, a recently reported “pan-beta” multi-epitope mRNA-LNP vaccine built around conserved B- and T-cell epitopes showed protective efficacy against divergent SARS-CoV-2 variants, illustrating that multi-epitope designs can translate into measurable protection even when neutralization breadth is challenged by antigenic drift (Vahed et al., 2025). In parallel, early clinical development programs such as CD40. Pan.CoV (EDC.Pan.CoV) indicate ongoing first-in-human evaluation of broad-spectrum concepts, reinforcing the field’s movement toward next-generation vaccine paradigms even though antigen formats and targeting strategies differ from epitope-string constructs (Nguema et al., 2024). Notably, the SARS-CoV-2 sequences used for epitope discovery were predominantly derived from ancestral or pre-Omicron diversity, and the conservation of the predicted epitopes should be explicitly evaluated against Omicron-era lineages in future analyses to contextualize relevance under contemporary antigenic drift. Taken together, these studies provide a comparative framework for interpreting our work as an epitope-level, RBD-focused construct that aims to extend breadth across pathogenic betacoronaviruses, while recognizing that definitive cross-protection requires experimental validation across representative sarbecovirus and merbecovirus systems (Cankat et al., 2023; Vahed et al., 2025).

### 3.12 Study limitations and future directions

This study relies on integrated *in silico* framework, including immunoinformatics and reverse vaccinology, molecular docking, molecular dynamics simulations, and immune simulations. Therefore, epitope selection, HLA population coverage, and immune simulation outputs should be interpreted as model-derived predictions that may not fully capture antigen processing, immunodominance hierarchies, epitope competition, or inter-individual variability in immune responsiveness. Although the construct was derived from conserved RBD regions of SARS-CoV, MERS-CoV, and SARS-CoV-2, cross-protection ultimately depends on whether these epitopes are naturally processed and presented in human APCs and whether they elicit protective antibody and T-cell responses *in vivo*. Docking and molecular dynamics provide supportive structural evidence for plausible Fab-like or BCR engagement and overall complex stability. However, ClusPro scores reflect comparative pose ranking rather than absolute binding affinity, and simulations cannot fully capture membrane-context, receptor avidity, glycan shielding, or other biophysical determinants that shape B-cell activation and neutralization. In addition, ongoing antigenic drift, including reduced neutralization against multiple Omicron subvariants after contemporary vaccination or prior infection, underscores the need to extend conservation analyses and experimental validation to currently circulating and emergent lineages.

Future studies should prioritize experimental validation by confirming construct expression and structural integrity, verifying epitope processing and presentation in relevant human APC systems, and measuring functional B- and T-cell responses using established immunoassays. *In vivo* evaluation, ideally in HLA-transgenic models, will be essential to evaluate immunogenicity, durability of memory, and breadth of protection across representative sarbecovirus- and merbecovirus-related challenges, while enabling iterative optimization of construct architecture and delivery strategy. These future steps will help align predicted breadth with empirical evidence and strengthen interpretation in the context of ongoing SARS-CoV-2 immune escape dynamics.

## 4 Conclusion

This study used an integrated immunoinformatics and reverse vaccinology pipeline to design and *in silico* evaluate a multi-epitope peptide vaccine construct derived from conserved RBD regions of SARS-CoV, MERS-CoV, and SARS-CoV-2, with broad predicted HLA coverage (93.28% estimated global coverage) and favourable model quality metrics. Docking and molecular dynamics supported a structurally plausible Fab-like engagement mode and complex stability, but these results reflect comparative, model-based structural feasibility rather than evidence of antigen specificity, cross-neutralization, or *in vivo* B-cell activation. Immune simulations suggested theoretical immunogenic potential under a prime–boost schedule, yet remain predictive and require experimental confirmation. Overall, the construct is a promising breadth-oriented *in silico* candidate, warranting validation of expression, epitope processing and presentation, functional B- and T-cell responses, and neutralization breadth against contemporary and emerging lineages to support pandemic preparedness.

## Data Availability

The original contributions presented in the study are included in the article/Supplementary Material. Further inquiries can be directed to the corresponding authors.
