# Supplementary material for: A multi-epitope pan-betacoronavirus vaccine construct predicted to induce broad-spectrum and durable immune responses: an immunoinformatics approach
Source: Front Bioinform. 2026 Mar 18;6:1784011. doi: 10.3389/fbinf.2026.1784011 (PMC13038886; doi:10.3389/fbinf.2026.1784011)
Supplement: Supplementary file 1 [file Supplementaryfile1.docx]

Supplementary Material

# Supplementary Table

**Supplementary Table 1**. Protein sequences of coronavirus S1 receptor-binding domains (RBDs) obtained from NCBI GenBank.

|  | **SARS-CoV** |  |
| --- | --- | --- |
| **Accession ID** | **Origin of Sequence** | **Host** |
| BAC81362.1 | Taiwan | *Homo sapiens* |
| AAP41037.1 | Canada: Toronto | *H. sapiens* |
| ADC35483.1 | China: Hongkong | *H. sapiens* |
| AAP50485.1 | German | *H. sapiens* |
| AAP72986.1 | Italy | *H. sapiens* |
| AAS10463.1 | China: Guangzhou, Guangdong | *H. sapiens* |
| AYV99817.1 | USA | *H. sapiens* |
|  | **MERS-CoV** |  |
| **Accession ID** | **Origin of Sequence** | **Host** |
| AGN70929.1 | Saudi Arabia | *H. sapiens* |
| UBS90429.1 | Kenya | *Camelus dromedarius* |
| QQK33722.1 | Tunisia | *C. dromedarius* |
| AWF93655.1 | Egypt | *Capra hircus* |
| ALU34110.1 | Spain | *C. dromedarius* |
| AJO62173.1 | Uni Emirate Arab | *C. dromedarius* |
| ALS20350.1 | Nigeria | *C. dromedarius* |
|  | **SARS-CoV 2** |  |
| **Accession ID** | **Origin of Sequence** | **Host** |
| QHR63270.2 | China: Wuhan | *H. sapiens* |
| UCF18109.1 | Canada | *H. sapiens* |
| UEJ84741.1 | Mali | *H. sapiens* |
| UEJ84776.1 | France | *H. sapiens* |
| UEO84925.1 | Russia | *H. sapiens* |
| UFO68656.1 | Brazil | *H. sapiens* |
| QPF17471.1 | USA: Pittsburgh Pennsylvania | *H. sapiens* |
| QSD16490.1 | Italy | *H. sapiens* |
| QJR85377.1 | Australia: Victoria | *H. sapiens* |

A total of 22 S1 RBD protein sequences representing three pathogenic coronaviruses—SARS-CoV, MERS-CoV, and SARS-CoV-2—were retrieved from the NCBI GenBank® database. The sequences were selected to reflect broad geographic diversity and multiple host species. For SARS-CoV, seven human-derived sequences originated from Taiwan, Canada (Toronto), Hong Kong, Germany, Italy, Guangzhou (China), and the USA. For MERS-CoV, seven sequences were included from Saudi Arabia (human host) and from animal reservoirs such as camels (Camelus dromedarius) in Kenya, Tunisia, Spain, the United Arab Emirates, and Nigeria, as well as goat-derived isolates from Egypt. For SARS-CoV-2, eight human sequences were sampled from China (Wuhan), Canada, Mali, France, Russia, Brazil, the USA (Pittsburgh, Pennsylvania), Italy, and Australia (Victoria). These datasets provide a representative cross-section of global sequence diversity across continents and hosts, ensuring the robustness of downstream multiple-sequence alignment and conserved-region analyses.

**Supplementary Table 2.** Docking scores of the post-refinement multi-epitope vaccine construct with the B-cell receptor (BCR) using the ClusPro server.

**
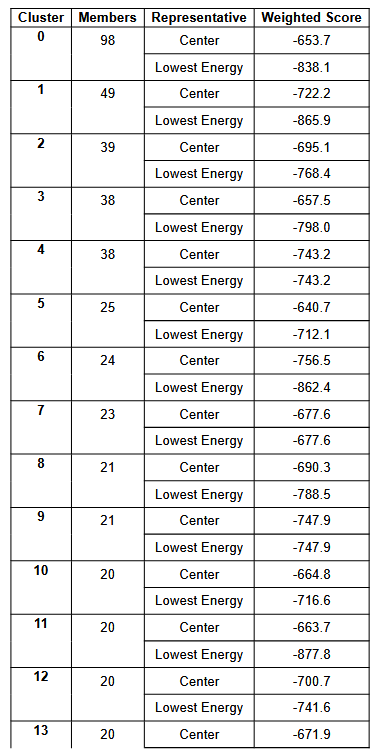

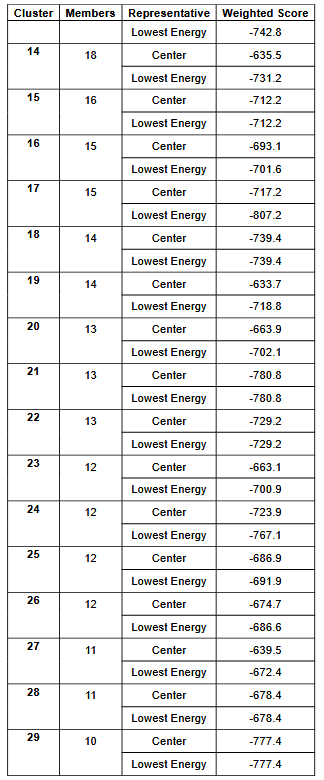
**

**Supplementary Table 3.** Details of interacting residues between the vaccine construct and the BCR light and heavy chains.


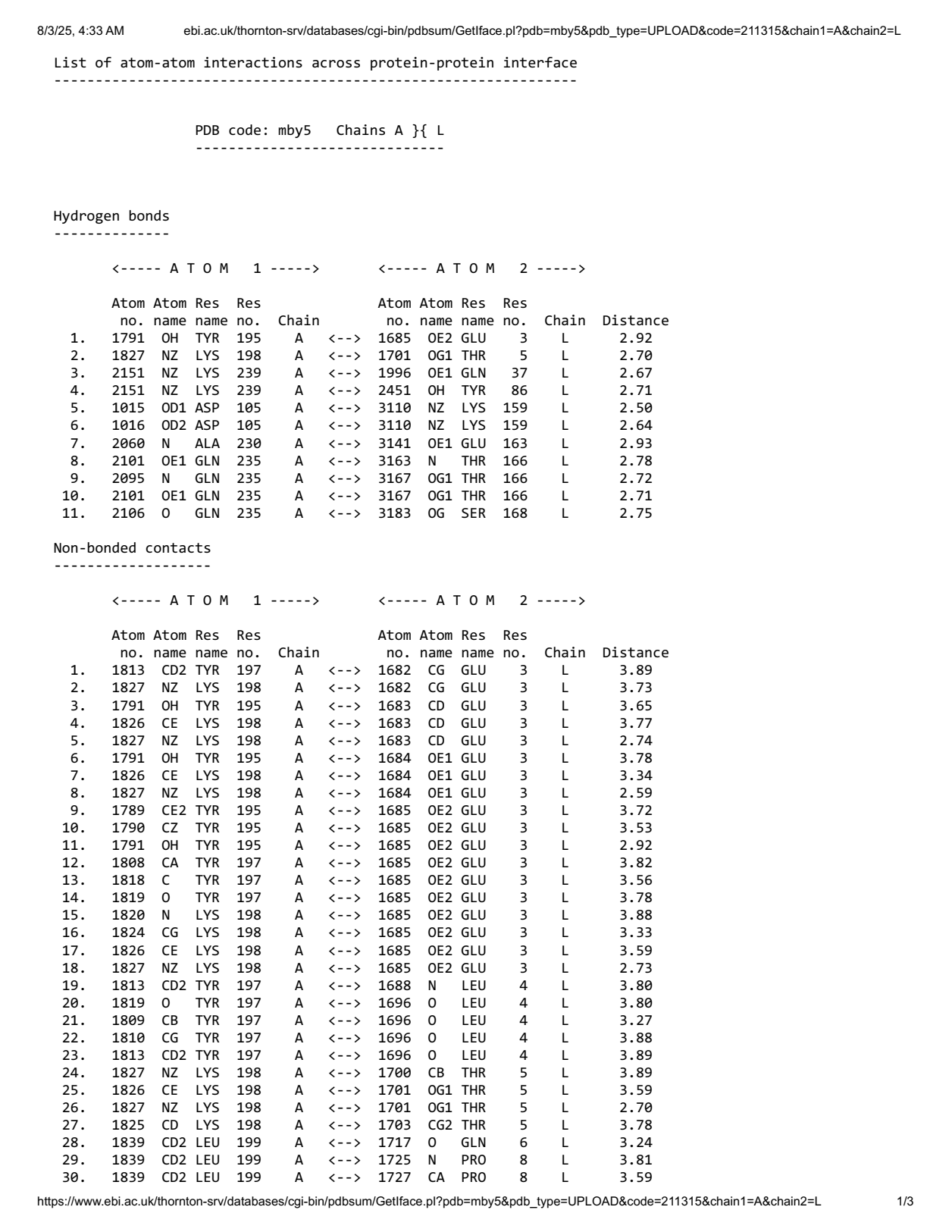


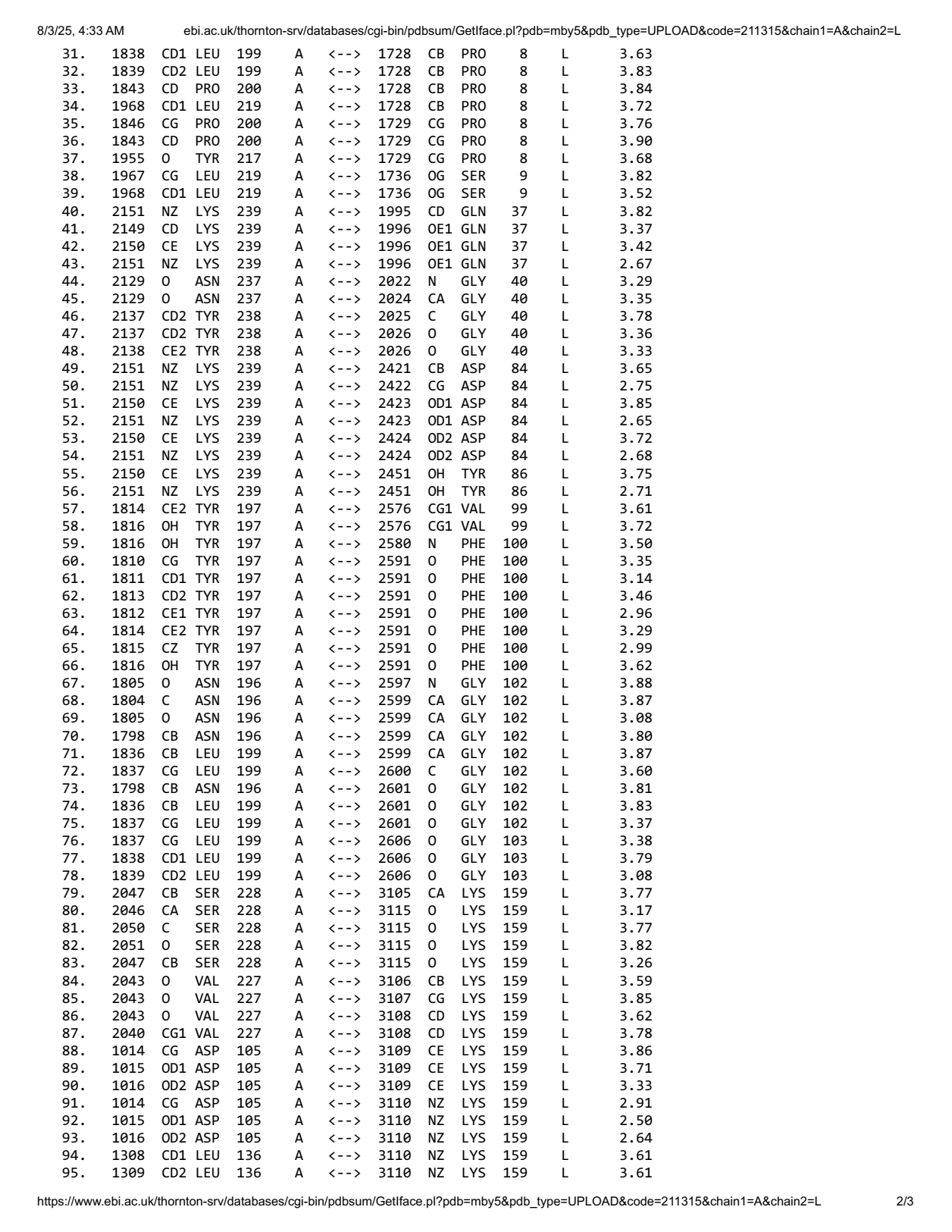


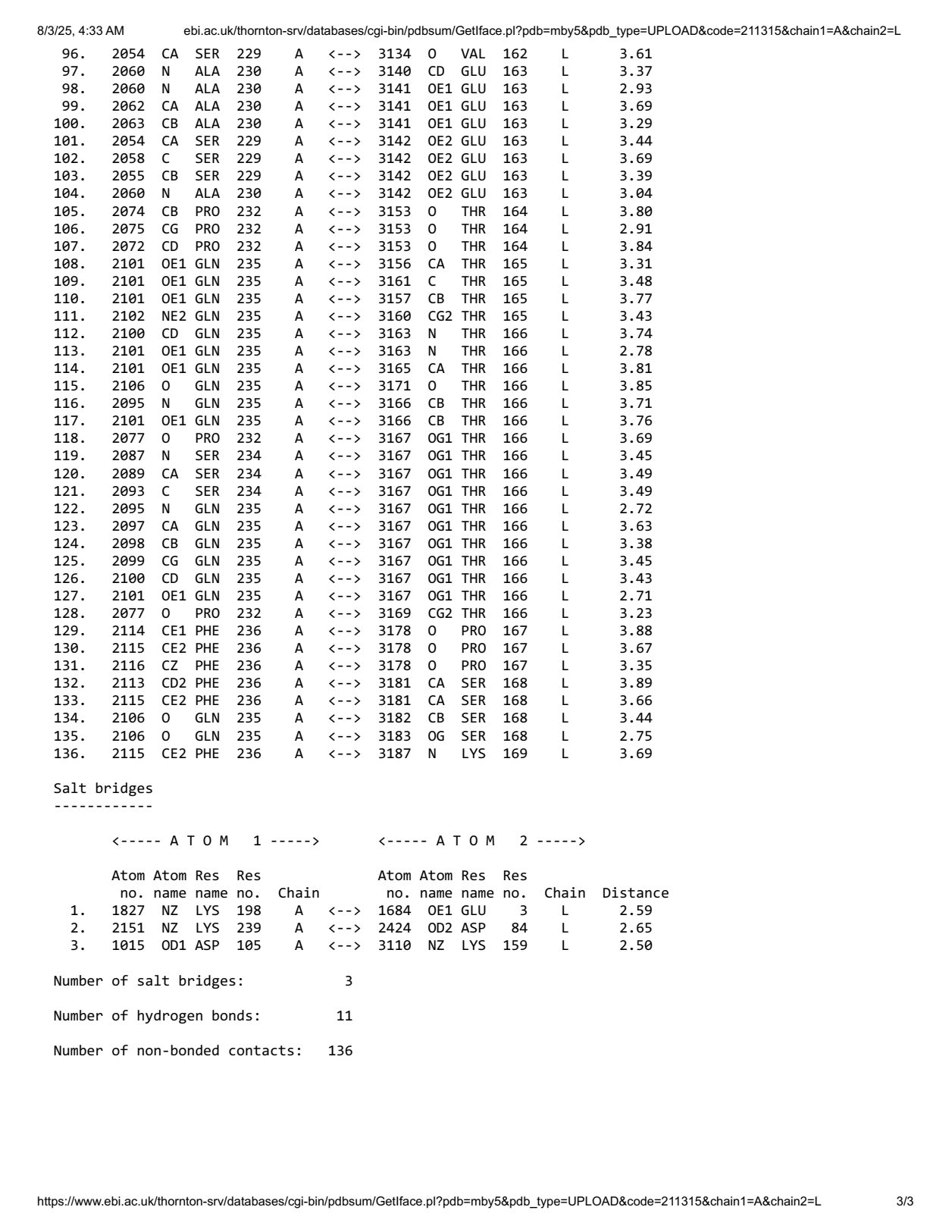


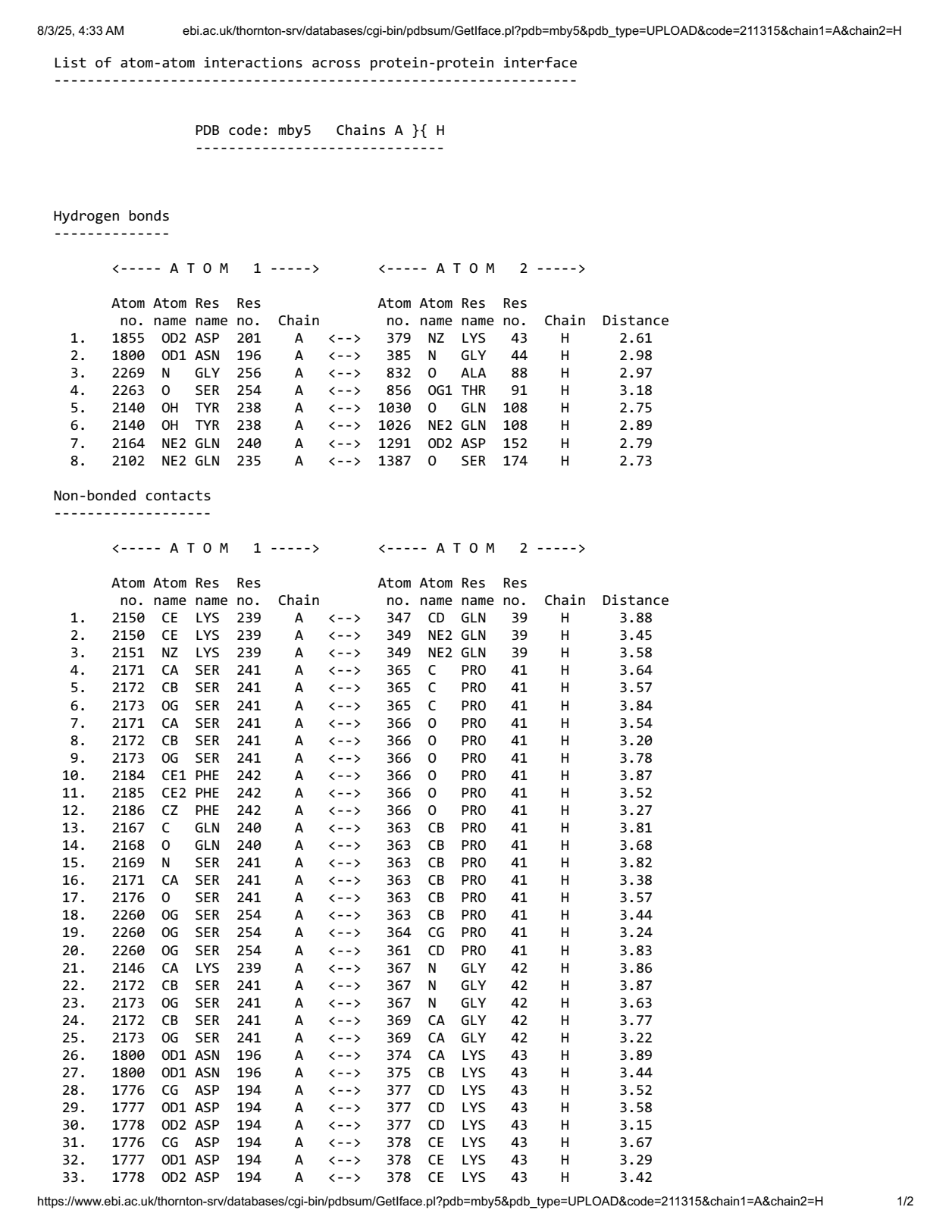


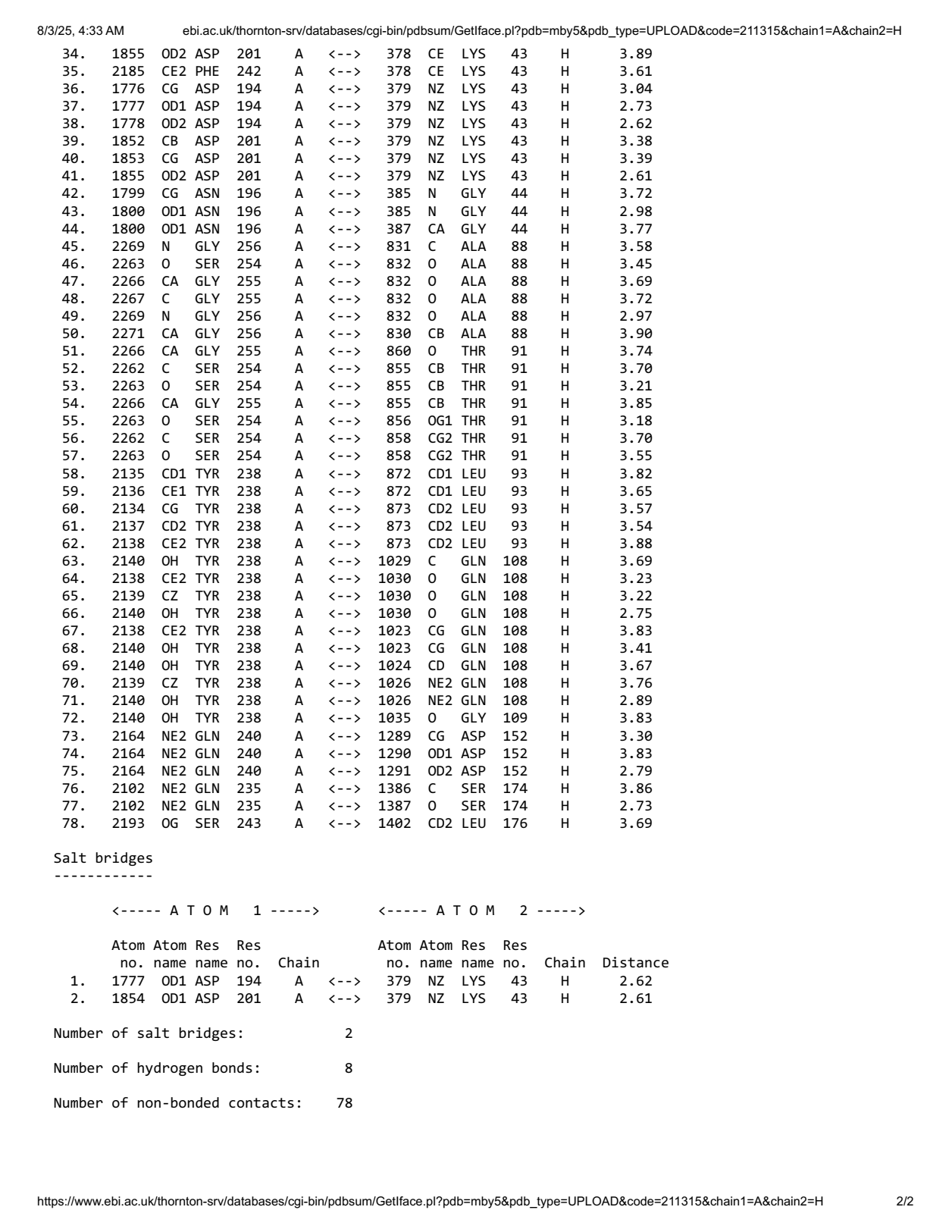


# Supplementary Figure

**Supplementary Figure 1.** Structural validation of the vaccine construct using a Ramachandran plot.

| 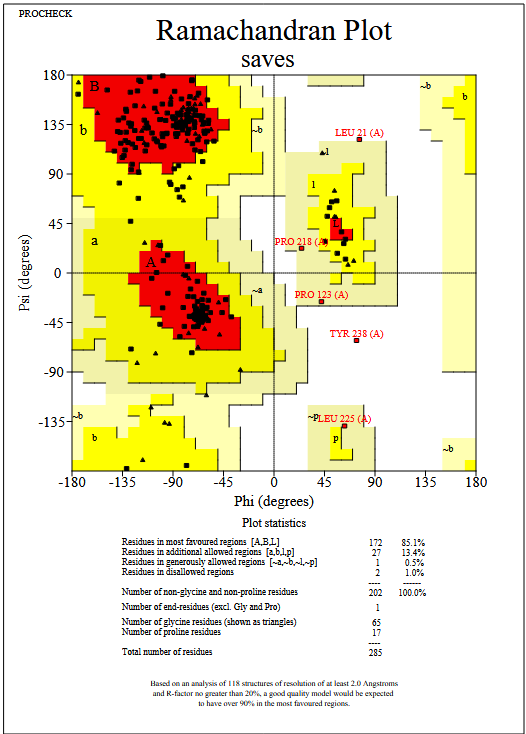 | *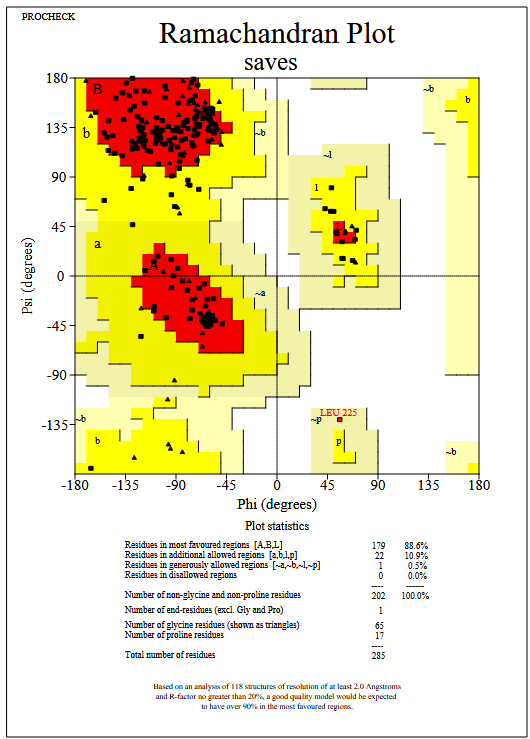* |
| --- | --- |
| (A) Pre-refinement | (B) Post-refinement |

The PROCHECK Ramachandran plot for the vaccine construct before refinement (A) indicates generally acceptable backbone stereochemistry, with most residues occupying the expected right-handed α-helix and β-sheet regions. Of the 202 non-glycine/non-proline residues assessed (including 1 end residue), 172 residues (85.1%) fall in the most favoured regions, 27 residues (13.4%) in additional allowed regions, 1 residue (0.5%) in generously allowed regions, and 2 residues (1.0%) in disallowed regions, while the construct contains 65 glycine and 17 proline residues that are evaluated separately due to their distinct conformational constraints. Overall, 98.5% of residues lie within allowed regions, supporting the plausibility of the initial model; however, the relatively modest proportion in the most favoured regions and the presence of two disallowed outliers suggest localized backbone strain, likely in flexible loop or linker segments, and justify subsequent refinement to improve stereochemical quality.

The PROCHECK Ramachandran plot for the vaccine construct after refinement (B) shows improved backbone stereochemistry, with residues clustering predominantly within the expected right-handed α-helix and β-sheet regions. Of the 202 non-glycine/non-proline residues evaluated (including 1 end residue, excluding Gly/Pro), 179 residues (88.6%) fall in the most favoured regions, 22 residues (10.9%) in additional allowed regions, 1 residue (0.5%) in generously allowed regions, and 0 residues (0.0%) in disallowed regions; the construct contains 65 glycine and 17 proline residues that are assessed separately due to their distinct conformational constraints. Overall, 100% of assessed residues lie within allowed regions, and the complete elimination of disallowed outliers indicates that refinement successfully reduced local backbone strain and improved stereochemical plausibility. Although the proportion in the most favoured regions remains slightly below the >90% benchmark often seen in high-resolution structures, this is still consistent with a well-behaved refined model, particularly for a flexible, linker-rich multi-epitope construct.
